# Case report: Treatment with Pien-Tze-Huang for prolonged positive SARS-CoV-2 test results in COVID-19 patients: A report of five cases

**DOI:** 10.3389/fmed.2022.860681

**Published:** 2022-08-09

**Authors:** Yujiao Zheng, Jinbo Cheng, Chengjuan Gu, Mingzhong Xiao, Zuoyu Shao, Linhua Zhao, Xiaolin Tong

**Affiliations:** ^1^College of Traditional Chinese Medicine, Anhui University of Chinese Medicine, Hefei, China; ^2^Xinqiao Hospital, Army Medical University, Chongqing, China; ^3^Shenzhen Hospital (Futian), Guangzhou University of Chinese Medicine, Guangzhou, China; ^4^Hubei Key Laboratory of Theoretical and Applied Research of Liver and Kidney in Traditional Chinese Medicine, Hepatic Disease Institute, Hubei Provincial Hospital of Traditional Chinese Medicine, Wuhan, China; ^5^Affiliated Hospital of Hubei University of Chinese Medicine, Wuhan, China; ^6^Guang'anmen Hospital, China Academy of Chinese Medical Sciences, Beijing, China

**Keywords:** COVID-19, RT-PCR, SARS-CoV-2, Chinese medicine, Pien-Tze-Huang

## Abstract

Coronavirus disease 2019 (COVID-19) has rapidly spread around the world since December 2019, becoming a global pandemic. Atypical cases of COVID-19, manifesting as prolonged positive SARS-CoV-2 test results during the convalescence period, have been encountered. These cases increase the difficulty of COVID-19 prevention and treatment. Here, we report five cases of COVID-19 patients who demonstrated prolonged positive SARS-CoV-2 tests after regular traditional Chinese medicine and western medicine treatments. After administration of Pien-Tze-Huang and cessation of previous treatments, SARS-CoV-2 tests results of the patients turned and remained negative. We believe the finding will contribute to a better understanding of atypical COVID-19 cases and hope to offer a potential therapy. Since this is a preliminary case series, larger-scale clinical trials are warranted.

## Introduction

The outbreak of coronavirus disease 2019 (COVID-19) was first reported in Wuhan, China, in December 2019 and subsequently rapidly spread around the world (1). It has been recognized as a global pandemic. Although the epidemic has been widely contained in China, the mechanism underlying COVID-19 has not yet been elucidated fully, and treatments for the disease are still being explored. It is noteworthy that some COVID-19 patients show repeat or prolonged positive reverse transcriptase–polymerase chain reaction (RT-PCR) test results during the convalescence period (2). A descriptive study performed in Wuhan showed that 30% of patients still had positive SARS-CoV-2 RT-PCR test results after treatment (3). These patients often manifest mild symptoms or are even asymptomatic, other than for positive RT-PCR test results, and their infectivity status is not clear, which can be a significant problem for disease prevention and control. Although there are many mature treatment plans for acute COVID-19 patients, it remains a clinical problem for convalescent patients with prolonged positive SARS-CoV-2 test results.

Traditional Chinese medicine (TCM) has a long history of being used to treat epidemic diseases, such as severe acute respiratory syndrome (SARS) and H1N1 influenza (4, 5). During the battle against COVID-19, TCM has also played a positive role in addressing the epidemic in China. A number of reports have shown that TCM treatment or TCM combined with western medicine was effective in treating mild, moderate, and severely ill COVID-19 patients as well as during convalescence (6–9).

Pien-Tze-Huang (PTH) is a famous TCM formula developed in the Ming Dynasty (around 1555 AD). Composed of *Radix et Rhizoma Notoginseng, Moschus, Calculus Bovis*, and *Snake Gall*, PTH has several functions, such as reducing fever, eliminating toxin, improving immunity, promoting blood circulation, and ameliorating pain (10). In clinical practice and animal tests, PTH has been applied in the treatment of autoimmune diseases, cancer, and various inflammatory diseases, including hepatitis, colitis, arthritis, and encephalomyelitis (11–14).

In our fight against COVID-19, we discovered that PTH had a positive effect on discharged patients who had had persistently positive SARS-CoV-2 test results. In this study, we report our experience of five of these SARS-CoV-2-positive patients whose test results became negative after PTH treatment.

## Case series presentation

The cases reported in this study were obtained from the medical records of confirmed COVID-19 patients who were transferred to designated hospitals after being hospitalized in Huoshenshan Hospital in Wuhan, from March 3 to April 19, 2020. Written informed consent in accordance with the Declaration of Helsinki has been obtained from all patients whose cases are reported in the study. During hospitalization, all these patients were treated using a systematic routine treatment plan that included TCM and western medicine for up to 4 weeks. The characteristics of the patients are listed in Tables 1, 2.

**Table 1:** Baseline characteristics of study patients.

	**Sex**	**Age (years)**	**Medical history**	**Epidemiological history**	**Symptoms**	**TCM**	**Western medicine**
Case 1	Male	74	Hypertension	None	Fever Cough	Huoxiang Zhengqi capsule Chinese herbal compound	Thymalfasin Recombinant human interferon α2b Arbidol hydrochloride Tablets
Case 2	Male	62	Hypertension	None	Fatigue Loss of appetite Fear of cold	Lianhua Qingwen capsule and Chinese herbal compound	Arbidol hydrochloride tablets, Interferon α2b Vitamin supplement, Intestinal flora regulation
Case 3	Male	61	Hypertension Type 2 diabetes Hypothyroidism	None	Fatigue Headache Dry cough Chest tightness Dyspnea	Lianhua Qingwen capsule and Chinese herbal compound	Arbidol hydrochloride tablets, Chloroquine phosphate Azithromycin, Thymalfasin Vitamin supplement
Case 4	Male	60	None	None	Fever Cough Fatigue	Lianhua Qingwen capsule and Chinese herbal compound	Arbidol hydrochloride tablets, Chloroquine phosphate, Interferon α2b, Thymalfasin, Vitamin supplement, Convalescent plasma therapy
Case 5	Male	45	Chronic nasopharyngitis Chronic bronchitis Hypertension	None	Fever Fear of cold Dry cough Fatigue Muscle pain	Chinese herbal compound	Pidotimod capsules, Compound glycyrrhizin tablets, Thymalfasin, Interferon α2b, Arbidol hydrochloride tablets

**Table 2:** Baseline laboratory tests and lung CT of study patients.

	**White blood cell count, × 10^**9**^ /L**	**Neutrophil count, × 10^**9**^ /L**	**Lymphocyte count, × 10^**9**^ /L**	**C-reactive protein, mg/L**	**Interleukin, pg/mL**	**CT results**
Case 1	5.3 (3.5.9.5)	3.3 (1.8.6.3)	1.5 (1.1,3.2)	1.24 (0,4)	2.32 (<7)	The markings of both lungs increased, and there were increasing high-density flocculent shadows in the outer zone of both lungs, most of which were ground-glass like changes.
Case 2	4.9 (3.5.9.5)	2.51 (1.8.6.3)	1.76 (1.1,3.2)	0.21 (0,4)	<1.5 (<7)	The fields of both lungs were clear, with a little more lung texture, and some cord-like shadow observed in both lungs
Case 3	3.8 (3.5.9.5)	2.01 (1.8.6.3)	1.21 (1.1,3.2)	0.7 (0,4)	–	Multiple high-density flocculent shadows were scattered in both lungs, mostly with ground glass changes, mainly distributed in the middle and outer zone of the lungs.
Case 4	6.5 (3.5.9.5)	4.35 (1.8.6.3)	1.44 (1.1,3.2)	0.87 (0,4)	<1.5 (<7)	The markings of both lungs were increased, and flocculent shadows with increasing density were seen in both lungs, showing ground glass-like changes.
Case 5	5.1 (3.5.9.5)	3.14 (1.8.6.3)	1.48 (1.1,3.2)	1.51 (0,4)	<1.5 (<7)	Flocculent ground glass shadow were observed in both lungs, with fuzzy boundaries, in the middle and outer zone of the lung field.

During the treatment and follow-up periods, throat-swab specimens were obtained from patients for SARS-CoV-2 detection by RT-PCR test. Patients who repeatedly had positive SARS-CoV-2 test results after completing treatment were enrolled in the study.

For the cases included in the study, PTH capsules (Zhangzhou Pien Tze Huang Pharmaceutical Co., Ltd., Zhangzhou, China) were administered orally for 14 days: two capsules were taken three times per day to address positive SARS-CoV-2 test results. During the PTH treatment period, at least two consecutive RT-PCR tests were performed on throat swab specimens obtained at a sampling interval of at least 24 h. Two consecutive negative RT-PCR test results were considered to indicate recovery, after which PTH administration was stopped. The PTH treatment process and follow-up are summarized in Table 3.

**Table 3:** PTH treatment for persistent positive SARS-CoV-2 test results in patients.

	**Date of diagnosis**	**Repeated positive test duration (days)**	**Other therapy**	**Date of PTH treatment**	**Dosage of PTH**	**Date of turning negative**	**Negative nucleic acid tests**	**Follow-up negative/positive**
Case 1	February 7, 2020	74	None	April 13, 2020	Two capsules three times a day	April 21, 2020	3 consecutive times	Negative
Case 2	February 26, 2020	55	None	April 18, 2020	Two capsules three times a day	April 21, 2020	5 consecutive times	Negative
Case 3	January 29, 2020	72	None	April 12, 2020	Two capsules three times a day	April 17, 2020	6 consecutive times	Negative
Case 4	February 11, 2020	60	None	April 12, 2020	Two capsules three times a day	April 14, 2020	5 consecutive times	Negative
Case 5	February 11, 2020	67	None	April 19, 2020	Two capsules three times a day	April 20, 2020	5 consecutive times	Negative

### Case 1

A 74-year-old male patient with a history of hypertension was evaluated for fever and cough lasting for 41 days. He was diagnosed with COVID-19 through SARS-CoV-2 detection and computed tomography (CT) of the lungs on February 7, 2020. He had received TCM treatment in another hospital before this hospitalization. However, his SARS-CoV-2 nucleic acid tests continued to be positive. During this hospitalization period, he had symptoms of fatigue, loss of appetite, and insomnia. He was started on TCM treatment, including Huoxiang Zhengqi capsule and Chinese herbal compound, and was also prescribed western medicine, including nasal oxygen, acetylcysteine, thymalfasin injection, recombinant human interferon α2b injection, umifenovir hydrochloride tablets (Arbidol®), and nifedipine sustained-release tablets.

After the above treatment, all the patient's symptoms were markedly alleviated, but SARS-CoV-2 test results on March 18, 19, 25, and 27, 2020, were still positive. Thymalfasin injection was added on March 26, 2020 to increase his immune response. SARS-CoV-2 test results on March 31 and April 1, 2020 were negative according to the medical records. Routine blood, C-reactive protein, and interleukin-6 test results on March 18, 2020 were normal. CT results on March 18, 2020 showed an increase in lung markings and increasing flocculent high-density shadows in the outer zones of both lungs, most of which were ground glass-like changes. He had no respiratory symptoms and no fever for more than a week, but SARS-CoV-2 test results became positive again when the patient was about to leave the hospital. Thus, the doctor decided to administer convalescent plasma therapy and interferon therapy. SARS-CoV-2 test results on April 2, 4, 6, and April 10, 2020 were still positive. He was therefore transferred to a designated hospital for observation.

There, PTH was prescribed from April 13, 2020, and other treatments were discontinued. With PTH treatment, consecutive RT-PCR tests for SARS-CoV-2 detection on April 21 and 24, 2020 were negative. The time elapsed from the onset of the disease to when the nucleic acid test results turned negative was 74 days. After 3 months of follow-up, the patient's SARS-CoV-2 test results were completely negative, and he no longer manifested any clinical symptoms or lung abnormalities on CT.

### Case 2

A 62-year-old male patient with a medical history of hypertension presented with fatigue, loss of appetite, and fear of cold. Through RT-PCR test for SARS-CoV-2 and CT of the lungs, he was diagnosed with COVID-19 on February 26, 2020 and admitted to hospital on March 7, 2020. He had received TCM treatment at another hospital before this hospitalization and did not have fever. However, SARS-CoV-2 tests continued to be positive. The TCM treatment included Lianhua Qingwen capsule and Chinese herbal compound, while western medicine included nasal oxygen, umifenovir hydrochloride tablets (Arbidol®), interferon α2b injection, vitamin supplements, intestinal flora regulation, amlodipine besylate tablets, and convalescent plasma therapy during this hospitalization period.

After the above treatment, all symptoms disappeared and CT scans of the lungs obtained on March 17, 2020 showed that the lung inflammation had improved. However, SARS-CoV-2 test performed on March 8, 11, 13, and 16, 2020 remained repeatedly positive. Routine blood, C-reactive protein, and interleukin-6 tests, performed on March 8, 2020, were normal. CT performed on March 14, 2020 showed that the fields of both lungs were clear, with a little more lung texture, and some cord-like shadow observed in both lungs. He had no respiratory symptoms. The attending doctor decided to administer convalescent plasma therapy on March 17, 2020. SARS-CoV-2 test result was negative after plasma therapy on March 21, 2020. However, it turned positive again 2 days later and continued to be positive in six subsequent tests performed until April 7, 2020. He was then transferred to a designated hospital for observation.

There, PTH was prescribed on April 18, 2020 and other treatments were discontinued. With PTH treatment, the patient's RT-PCR tests performed on April 21 and 24, 2020 were negative. A follow-up on July 20, 2020 also yielded a negative SARS-CoV-2 test and normal lung CT findings. The time elapsed from the onset of the disease to when the nucleic acid test results turned negative was 55 days. After 3 months of follow-up, the patient's SARS-CoV-2 test results were completely negative, and he had no further clinical symptoms or lung problems on CT.

### Case 3

A 61-year-old male patient with a history of hypertension, type 2 diabetes, and hypothyroidism, presented with fatigue, headache, dry cough, chest tightness, and dyspnea on January 29, 2020. His RT-PCR test for SARS-CoV-2 was positive and CT of the lungs showed infection in both on February 26, 2020. After being diagnosed with COVID-19, he received western medicine treatment in another hospital before this hospitalization. Dyspnea was relieved, but he still had a paroxysmal dry cough. During this hospitalization period, he received TCM combined with western medicine. TCM treatment included Lianhua Qingwen capsule and Chinese herbal compound. Western medicine treatment included umifenovir hydrochloride tablets (Arbidol®) tablets, chloroquine phosphate combined with azithromycin, thymalfasin injection, vitamin supplements, nifedipine sustained-release tablets, acarbose, and levothyroxine sodium (Euthyrox®).

During hospitalization, SARS-CoV-2 test results of the patient on March 7, 11, 13, 14, and 17, 2020 were persistently positive. Thus, PTH was administered, and other treatments were discontinued. SARS-CoV-2 test result was negative on March 25, 2020. However, it turned positive again 2 days later and continued to be positive in seven subsequent tests, even though the doctor decided to administer convalescent plasma on April 8, 2020. He was therefore transferred to a designated hospital for observation due to prolonged positive SARS-CoV-2 test results.

Routine blood and C-reactive protein tests performed on March 4, 2020 were normal. CT performed on March 6, 2020 showed multiple high-density flocculent shadows scattered in both lungs, mostly involving ground glass changes and mainly distributed in the middle and outer zones of the lungs.

With PTH treatment from April 12, 2020, the patient's RT-PCR test results on April 17 and 19, 2020 were negative, and follow-up RT-PCR test results on May 29 and July 24, 2020 were also negative. The time elapsed from disease onset to a negative test result was 72 days. After 3 months of follow-up, SARS-CoV-2 test results became completely negative, and the patient manifested no clinical symptoms or lung CT problems.

### Case 4

A 60-year-old male patient with no significant medical history was diagnosed with COVID-19 and presented to the hospital with fever, cough, and fatigue and had positive RT-PCR test results for SARS-CoV-2 on February 11, 2020. He had received TCM treatment at another hospital before this hospitalization. SARS-CoV-2 test results were negative on February 21, 25, and 28, 2020. However, it turned positive again on March 7, 2020 and continuously to be positive. During this hospitalization period, he was depressed and had little appetite. TCM combined with western medicine was administered. TCM treatment included Lianhua Qingwen capsule and Chinese herbal compound. Western medicine treatment included umifenovir hydrochloride tablets (Arbidol®) tablets, chloroquine phosphate, interferon α2b injection, thymalfasin injection, vitamin supplements, acetylcysteine, and convalescent plasma therapy.

During hospitalization, the patient's SARS-CoV-2 test results were continuously positive, even though other symptoms were improved significantly. Routine blood, C-reactive protein, and interleukin-6 tests on March 21, 2020 were normal. CT results obtained on March 24, 2020 showed that the markings in both lungs had increased, and an increasing density of flocculent shadows were seen in both lungs, showing ground glass-like changes. He had no respiratory symptoms. The attending doctor decided to administer convalescent plasma therapy on March 28, 2020. After plasma infusion, SARS-CoV-2 test results on April 2 and 5, 2020 became negative. However, it turned positive again 2 days later. He was consequently transferred to a designated hospital for observation.

He started PTH treatment from April 12, 2020 and stopped other treatments. RT-PCR test results were negative on April 14 and 17, 2020, which indicated recovery. Follow-up SARS-CoV-2 test results on June 4, 2020 were also negative. The time elapsed from disease onset to a negative test result was 60 days. After 3 months of follow-up, SARS-CoV-2 test results of the patient were completely negative, and he manifested no clinical symptoms or lung abnormalities on CT.

### Case 5

A 45-year-old male patient with a medical history of chronic nasopharyngitis, chronic bronchitis, and hypertension was diagnosed with COVID-19 and had repeat positive SARS-CoV-2 test results on February 12, 2020. He presented with fever, fear of cold, dry cough, fatigue, and muscle pain. After being admitted to the hospital, he was administered Chinese herbal compound and western medicine treatments, including nasal oxygen, Pidotimod capsules, compound glycyrrhizin tablets, thymalfasin for injection, interferon α2b injection, and umifenovir hydrochloride tablets (Arbidol®) tablets.

After treatment, the clinical symptoms disappeared. However, SARS-CoV-2 test results were repeatedly positive. Routine blood, C-reactive protein, and interleukin-6 tests performed on March 10, 2020 were normal. CT results showed flocculent ground glass shadows in both lungs, with fuzzy boundaries, which were present in the middle and outer zones of the lung field. He had no respiratory symptoms. CT of the lungs performed on March 10, 2020 showed that the lung inflammation improved a lot than before. SARS-CoV-2 test result was negative on April 9, 2020. However, it turned positive again a day later. He was therefore transferred to a designated hospital for observation.

PTH was administered to the patient from April 19, 2020, while other treatments were stopped. On April 20 and 24, 2020, SARS-CoV-2 RT-PCR test results were negative. Follow-up on July 3, 2020 again showed a negative SARS-CoV-2 test result. The time elapsed from the onset of the disease to when the nucleic acid test results turned negative was 67 days. After 3 months of follow-up, SARS-CoV-2 test results remained negative, and he had no clinical symptom or lung abnormalities on CT.

## Discussion

In the prevention and control of COVID-19, repeat or prolonged positive SARS-CoV-2 test result has caused widespread concern. Many studies have found that the incidence of positive SARS-CoV-2 test results in recovered COVID-19 patients after treatment is relatively high, which makes it difficult to evaluate whether patients have been completely cured (15). Additionally, this phenomenon has caused concern about whether individuals with a positive SARS-CoV-2 test result are contagious. A case study of prolonged infectious SARS-CoV-2 shedding from an asymptomatic immunocompromised patient reported that no infectious SARS-CoV-2 could be cultured, although the virus replicated actively, based on sgRNA presence (16). Some studies have demonstrated that the cause for re-positive tests for SARS-CoV-2 during the recovery period is false-positive RT-PCR results, considering the low sensitivity of RT-PCR tests for the diagnosis of COVID-19 in nasopharyngeal samples (17). Other scientists and some infection control guidelines assume that the reason for recurrent or prolonged positive SARS-CoV-2 test results in recovered patients may be the presence of viral antibody residues in the body or delayed clearance of viral RNA (18–20). Reactivation of the virus after treatment or exposure to a contaminated environmental surface during the recovery period are also reasons for repeat positive SARS-CoV-2 test results (21, 22). To date, no cases of infection among the population who have been in contact with patients with repeat or prolonged positive test results have been reported; thus, the infectivity of the group is unknown. Given that the RT-PCR test cannot distinguish active virus from inactive virus, it is likely that the viral RNA detected in repeat positive COVID-19 patients is inactivated; therefore, such positive test results may not represent reactivation or re-infection (23). Although the infectivity of these patients is unknown, it is necessary to eliminate the virus thoroughly and promote a permanent negative status for SARS-CoV-2 test results to help control the epidemic and alleviate fear among the population.

In this case series, we reported five cases of COVID-19 patients who had prolonged positive SARS-CoV-2 tests after typical TCM and western medicine treatments. Since SARS-CoV-2 testing at that time was hampered by factors such as limited medical resources, we did not perform SARS-CoV-2 tests every day, but every 2–5 days. By administering PTH and stopping regular treatments, the SARS-CoV-2 tests results became consistently negative. As a Chinese herbal compound that has the TCM function of clearing heat and detoxification, PTH may have an effect on clearing residual virus and improving immunity in COVID-19 patients. *Radix et Rhizoma Notoginseng*, an ingredient of PTH, can promote blood circulation and remove stasis according to TCM theory. Based on screening studies, some reports have stated that *Radix et Rhizoma Notoginseng* is a potential Chinese medicine against SARS-CoV-2 infection (24). It has been demonstrated that the main active compound of *Radix et Rhizoma Notoginseng*, Notoginsenoside R1, has anti-inflammatory and lung, liver, and gastrointestinal protective effects (25, 26). *Moschus* is the natural secretion of the musk deer. The primary component of *Moschus*, muscone, has markedly anti-inflammatory effects and has been widely applied to various inflammatory diseases (27). *Calculus Bovis* is commonly used in the treatment of fever, convulsion, and stroke. It has a wide range of pharmacological effects on the respiratory, nervous, cardiovascular, digestive, and immune systems (28). According to a literature analysis of Mongolian medicine prescriptions for preventing COVID-19, *Moschus* and *Calculus Bovis* are frequently used medicines in this clinical application (29). *Snake Gall* can also clear heat and remove toxicity according to TCM theory. Its main component, sodium taurodeoxycholate, has been found to have anti-inflammatory, immune regulatory, anti-oxidative, and neuroprotective effects (30, 31). To date, no pharmacological study has directly demonstrated the effect of PTH against COVID-19 although some studies have found that PTH can improve immunity by regulating Th1 and Th17 cells (32). T-cell-mediated adaptive immune responses are essential for clearance of viral antibodies and long-term antiviral immunity. The balance of Th1 and Th17 can be a primary target in SARS-CoV-2 infection and could contribute to COVID-19 treatment (33). Based on the above pharmacological mechanisms of PTH and its ingredients, we speculated that the mechanism by which PTH turns SARS-CoV-2 tests from positive to negative may include improving immunity by regulating T-cell responses and alleviating inflammation, which could help clear residual virus. However, normal host immunity also provides some antiviral ability, which may explain why SARS-CoV-2 tests of these patients turned negative. Thus, further studies are needed to explore whether PTH has direct anti-SARS-CoV-2 effects. Moreover, before being administered PTH treatment, the patients underwent regular TCM and western medicine interventions. Although the nucleic acid test did not become permanently negative thereafter, the previous interventions may also have contributed to facilitating recovery of the patients.

In addition, PTH has been widely used in clinical practice in China with good efficacy in infectious diseases. This has been included in the *Expert Guidance on TCM Treatment of Ebola Hemorrhagic Fever (first edition)* (34) and *Guidelines for Diagnosis and Treatment of Dengue fever (2014 2nd edition)* (35). Our study reported the effect of PTH on COVID-19 patients with repeat or persistent positive RT-PCR test results, which have not been reported previously. Nevertheless, this case series simply reflects our observations, and more data are needed to prove the efficacy of PTH treatment in such patients. Since the study was only a preliminary case series, its sample size was small and the sample features were concentrated, which were mostly elderly men. Therefore, a future larger-scale, randomized controlled clinical trial is necessary to confirm our results. Furthermore, *in vitro* and *vivo* experiments are needed to verify the role of PTH in COVID-19 treatment, which is our next work in the future.

## Conclusions

Our preliminary observations in five COVID-19 cases demonstrated that PTH may be a key player in the treatment of this disease in patients who present with persistent positive SARS-CoV-2 tests. Further investigation of the underlying pharmacological mechanisms and larger-scale randomized controlled trials are needed in the future.

## Data availability statement

The original contributions presented in the study are included in the article/supplementary material, further inquiries can be directed to the corresponding author/s.

## Ethics statement

Written informed consent was obtained from the individual(s) for the publication of any potentially identifiable images or data included in this article.

## Author contributions

XT and LZ designed the study. JC, ZS, and MX provided the original data of the clinical cases. YZ, CG, and MX wrote the manuscript and analyzed the data. All authors agree to be accountable for all aspects of work ensuring integrity and accuracy.

## Funding

This work was financially supported by the National Key Research and Development Program of China (2018YFC2000500 and 2020YFC0861100) and 2021 High-level Talent Introduction Scientific Project of Anhui University of Chinese Medicine.

## Conflict of interest

The PTH capsules used in this study were provided by Zhangzhou Pien Tze Huang Pharmaceutical Co., Ltd. The authors declare that the research was conducted in the absence of any commercial or financial relationships that could be construed as a potential conflict of interest.

## Publisher's note

All claims expressed in this article are solely those of the authors and do not necessarily represent those of their affiliated organizations, or those of the publisher, the editors and the reviewers. Any product that may be evaluated in this article, or claim that may be made by its manufacturer, is not guaranteed or endorsed by the publisher.
